# Clinicopathological characteristics and outcomes of anti-neutrophil cytoplasmic autoantibody-related renal vasculitis with hyperuricemia: a retrospective case-control study

**DOI:** 10.1038/s41598-021-81664-z

**Published:** 2021-01-21

**Authors:** Ruiqiang Wang, Dongyue An, Yunqi Wu, Pupu Ma, Yuanyuan Guo, Lin Tang

**Affiliations:** Department of Nephrology, The First Affiliated Hospital of Zhengzhou University, Zhengzhou, 450052 Henan Province China

**Keywords:** Diseases, Nephrology

## Abstract

The objective of the study was to evaluate the clinicopathological characteristics and investigate the clinical determinants of patient and renal survival in the first 12 months after diagnosis in anti-neutrophil cytoplasmic antibody (ANCA)-associated renal vasculitis (AAV) patients with hyperuricemia. This was a retrospective case-control study in patients with AAV-related renal injury in the First Affiliated Hospital of Zhengzhou University from January 2014 to April 2019. Patients who met the study criteria were divided into two groups: patients without hyperuricemia (n = 92) and patients with hyperuricemia (n = 55). Participants were followed-up for 12 months, and progressing to end-stage renal disease (ESRD) and death was treated as the endpoint event. We found that the level of serum creatinine was an independent risk factor for hyperuricemia, and the level of serum uric acid was an independent risk factors for renal survival and patient survival in ANCA-associated renal vasculitis patients. The crescents formation and the proportion of fibrous crescent likely contributed to severe clinical characteristics and renal pathological changes in ANCA-associated renal vasculitis patients with hyperuricemia. Hyperuricemia has an important influence on the progression of ANCA-associated renal vasculitis. A good control of serum uric acid may improve the prognosis.

## Introduction

Anti-neutrophil cytoplasmic antibody (ANCA)-associated vasculitis (AAV) is a small-vessel vasculitis characterized by necrotizing inflammation of small vessels (including small arteries, arterioles, capillaries and venules) in conjunction with ANCA. AAV is also a chronic autoimmune disease associated with multisystem dysfunction, and renal involvement is one of the most common manifestations which are closely associated with the outcomes. AAV patients with renal injury are increasingly recognized as a life-threatening disease, with about 20–25% of patients progressing to the end-stage renal disease (ESRD) within several years^1^.

Serum uric acid (SUA) is the final enzymatic product of purine metabolism of bodies. Abnormal nucleic acid metabolism and/or dysuric acid excretion in vivo caused by any reason can affect the level of blood uric acid, leading to the prevalence of hyperuricemia. Hyperuricemia has been found to be associated with damage of impartment organs and results in many diseases such as hypertension^2^, metabolic syndrome^3^, atherosclerosis^4^, myocardial infarction^5^, diabetes mellitus^6^, stroke^7^ and so on. Studies have shown that hyperuricemia is an independent risk factor in the progression of IgA nephropathy^8,9^. Hyperuricemia plays an important role in the development or progression of acute kidney injury (AKI) and chronic kidney disease (CKD) although there is no clear cutoff uric acid value associated to the risk for kidney damage^10,11^. However, few studies were reported about the detailed characteristics of ANCA-associated renal vasculitis patients with hyperuricemia. In this single-center retrospective study, we investigated the clinicopathological characteristics and the clinical determinants of patient and renal survival in ANCA-associated renal vasculitis patients.

## Results

### Demographic and clinical characteristics

A total of 147 ANCA-associated renal vasculitis patients (92 without hyperuricemia and 55 with hyperuricemia) were enrolled. There were 72 (48.98%) with hypertension, 12 (8.16%) with diabetes mellitus and 10 (6.80%) with cardiovascular diseases at diagnosis. Among 55 patients with hyperuricemia, there were 27 (49.09%) males and 28 (50.91%) females. No significant difference was observed between genders. Table 1 shows the baseline clinical characteristics and laboratory parameters at diagnosis in the two groups. Compared with the patients without hyperuricemia, the levels of estimated glomerular filtration rate (eGFR) and C-reaction protein (CRP) were significantly lower in patients with hyperuricemia, while the level of systolic blood pressure, diastolic blood pressure, serum creatinine, total cholesterol and triglycerides were significantly higher.

**Table 1:** Demographics and clinical characteristics of 147 ANCA-associated renal vasculitis patients with and without hyperuricemia.

Parameter	Without HUA (n = 92)	With HUA (n = 55)	*P* value
Age (years)	60.17 ± 11.14	58.36 ± 12.51	0.364
**Gender (male/female, n)**			
Male	49 (53.26)	27 (49.09)	0.626
Female	43 (46.74)	28 (59.91)	
**Hypertension, n (%)**			
No	51 (55.43)	24 (43.64)	0.168
Yes	41 (44.57)	31 (56.36)	
**Diabetes mellitus, n (%)**			
No	82 (89.13)	53 (96.36)	0.122
Yes	10 (10.87)	2 (3.64)	
**Cardiovascular diseases, n (%)**			
No	85 (92.39)	52 (94.55)	0.617
Yes	7 (7.61)	3 (5.45)	
Systolic BP (mmHg)	142.43 ± 28.63	154.35 ± 26.82	0.014
Diastolic BP (mmHg)	89.41 ± 10.15	93.20 ± 10.08	0.030
Uric acid (µmol/L)	276.35 ± 68.81	459.33 ± 77.43	< 0.001
Serum creatinine (µmol/L)	227.08 ± 152.68	330.64 ± 175.37	< 0.001
eGFR (ml/min/1.73 m2)	36.80 (16.02–49.27)	25.27 (9.91, 34.00)	< 0.001
Hemoglobin (g/L)	90.29 ± 16.08	93.14 ± 20.68	0.353
Serum albumin (g/L)	34.65 ± 35.38	33.81 ± 4.84	0.861
24 h-proteinuria (g)	2.01 (0.91–2.41)	2.18 (1.04–2.29)	0.139
Urine RBC counts (RBCs/HPF)	249.11 (14.25–354.50)	240.82 (38.00, 322.00)	0.421
CRP (mg/L)	56.17 (5.05–99.50)	33.98 (2.40, 54.70)	0.038
ESR (mm/h)	74.32 ± 40.53	75.15 ± 36.69	0.901
Complement C3 (mg/L)	1.07 (0.89–1.29)	1.10 (0.92–1.26)	0.917
Complement C4 (mg/L)	0.27 (0.22–0.31)	0.30 (0.24, 0.35)	0.121
Total cholesterol (mmol/L)	4.17 ± 1.14	4.76 ± 1.39	0.006
Triglycerides (mmol/L)	1.22 (0.90–1.49)	1.50 (1.05–1.70)	0.024
HDL-C (mmol/L)	1.13 (0.81–1.40)	1.41 (0.85–1.30)	0.895
LDL-C (mmol/L)	2.69 ± 1.19	2.84 ± 1.06	0.438
Anti-MPO (U/mL)	791.14 ± 172.40	584.89 ± 450.74	0.387
Anti-PR3 (U/mL)	49.68 (3.00–10.75)	72.36 (5.00–14.00)	0.146
BVAS	17.80 (15.00–21.00)	17.95 (15.00–20.00)	0.717

Values for continuous variables were given as the mean ± standard deviation (for normally distributed data) or median (interquartile range [IQR]; for nonnormally distributed data). Values for categorical variables were given as number or number (percentage).

*HUA* hyperuricemia, *eGFR* estimated glomerular filtration rate, *BP* blood pressure, *RBC* red blood cell, *ESR* erythrocyte sedimentation rate ,*CRP* C reactive protein, *HDL-C* high-density lipoprotein cholesterol, *LDL-C* low-density lipoprotein cholesterol, *Anti-MPO* myeloperoxidase antibody, *Anti-PR3* protease 3 antibody, *BVAS* Birmingham Vasculitis Activity Score.

*P* value: comparison between two groups. *P* < 0.05 was considered significant.

There were no significant differences in age, patients with hypertension at diagnosis, patients with diabetes mellitus at diagnosis, patients with diabetes mellitus at diagnosis, patients with cardiovascular diseases at diagnosis, ESR, hemoglobin, serum albumin, 24 h-proteinuria, urine RBC counts, complement C3, complement C4, HDL-C, LDL-C, anti-MPO, anti-PR3 and BVAS between the patients in two groups (Table 1).

### Proportion of patients with hyperuricemia for each CKD stage

**Figure 1:**
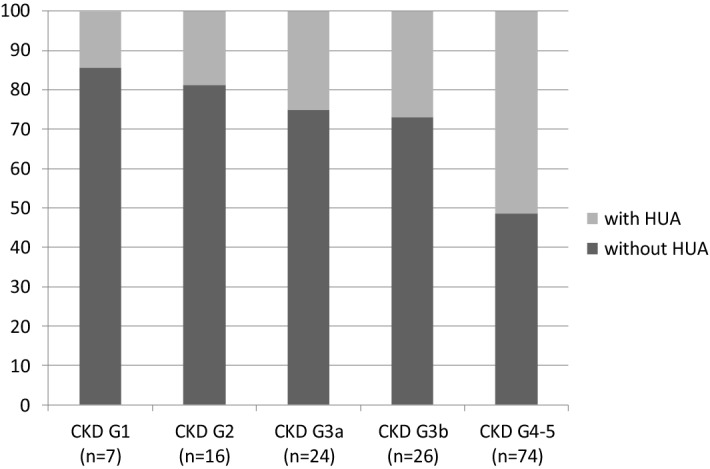
Proportion of patients with HUA for each CKD stage. The proportion of patients with HUA was 14.29% in CKD stage G1, 18.75% in CKD stage G2, 25.00% in CKD stage G3a, 26.92% in CKD stage G3b, and 51.35% in CKD stage G4-5. This was a significant difference among the CKD stages (*P* = 0.013).

The proportion of patients with hyperuricemia was 14.29% (1/7 patients) in CKD stage G1, and increased significantly with increasing CKD stage (Fig. 1). There was a significant difference among the CKD stages (*P* = 0.013).

### Pathological characteristics

The proportion of normal glomeruli (31.20% ± 21.81% vs. 45.02% ± 27.40%, *P* = 0.001) was worse in patients with hyperuricemia than patients without hyperuricemia, while the proportion of crescent (55.12% ± 22.73% vs. 46.22% ± 27.91%,* P* = 0.047), the proportion of fibrous crescent (33.75% ± 26.06% vs. 19.97% ± 24.29%, *P* = 0.001), tubular atrophy integral (0.67 (0.00, 1.00 vs. 0.41 (0.00, 1.00), *P* = 0.031 ) and interstitial fibrosis integral (1.51 (1.00, 3.00) vs. 1.09 (0.00, 2.00), *P* = 0.020) were more severe. No difference was observed in cellular crescent, fibrocellular crescent, fibrinoid necrosis, global glomerulosclerosis, destruction of Bowman's BM and microangiopathic lesions between two groups (Table 2).

**Table 2:** Pathologic characteristics of AAV-related renal involvement patients with or without hyperuricemia at the time of biopsy.

Parameter	Without HUA (n = 92)	With HUA (n = 55)	*P* value
Total number of glomeruli (n)	24.03 ± 12.34	26.65 ± 13.19	0.226
Normal glomeruli (%, (mean ± SD))	45.02% ± 27.40%	31.20% ± 21.81%	0.001
Crescent (%, (mean ± SD))	46.22% ± 27.91%	55.12% ± 22.73%	0.047
Cellular crescent (%, (median, IQR))	17.27% (0.00, 26.24%)	12.82% (0.00, 21.05%)	0.437
Fibrocellular crescent (%, (median, IQR))	9.30% (0.00, 15.52%)	8.54% (0.00, 12.00)	0.713
Fibrous crescent (%, (mean ± SD))	19.97% ± 24.29%	33.75% ± 26.06	0.001
Fibrinoid necrosis (%, (mean ± SD))	2.23% ± 11.32%	0.23% ± 0.98%	0.096
Global glomerulosclerosis (%, (median, IQR))	8.89% (0.00, 11.71%)	13.69% (0.00, 21.05%)	0.057
Destruction of Bowman's BM (%, (mean ± SD))	22.94% ± 19.40%	24.83% ± 17.14%	0.552
Tubular atrophy integral	0.41 (0.00, 1.00)	0.67 (0.00, 1.00)	0.031
Interstitial fibrosis integral	1.09 (0.00, 2.00)	1.51 (1.00, 3.00)	0.020
Interstitial inflammatory cell infiltration integral	2.11 (2.00, 3.00)	2.20 (1.00, 3.00)	0.582
**Microangiopathic lesions, n (%)**			
No	81 (88.04)	51 (92.73)	0.364
Yes	11 (11.96)	4 (7.27)	

Values for continuous variables were given as the mean ± standard deviation (for normally distributed data) or median (interquartile range [IQR]; for nonnormally distributed data). The semi-quantitative scoring system was used to analyze tubulointerstitial lesions. Values for categorical variables were given as number or number (percentage).

*P* value: comparison between two groups. *P* < 0.05 was considered as significant. HUA, hyperuricemia; BM, basement membrane.

### Hyperuricemia-associated influencing factors in patients with AAV-related renal vasculitis

The influencing factors were analyzed in patients with AAV-related renal vasculitis. Results of univariate logistic regression analysis showed that systolic blood pressure (*OR* = 1.015, *95% CI* = 1.003–1.027, *P* = 0.015), diastolic blood pressure (*OR* = 1.037, *95% CI* = 1.003–1.072, *P* = 0.033), serum creatinine (*OR* = 1.004, *95% CI* = 1.002–1.006, *P* = 0.001), total cholesterol (*OR* = 1.457, *95% CI* = 1.104–1923, *P* = 0.008), triglycerides (*OR* = 1.943, *95% CI* = 1.091–3.460, *P* = 0.024) , the proportion of crescent (*OR* = 1.013, *95% CI* = 1.000–1.027, *P* = 0.049), the proportion of fibrous crescent (*OR* = 1.021, *95% CI* = 1.008–1.035, *P* = 0.002), tubular atrophy integral (*OR* = 1.555, *95% CI* = 1.003–2.409, *P* = 0.048 ) and interstitial fibrosis integral (*OR* = 1.428, *95% CI* = 1.046–1.948, *P* = 0.025) were positively correlated with hyperuricemia. eGFR (*OR* = 0.977, *95% CI* = 0.961–0.994, *P* = 0.007), CRP (OR = 0.990,95% CI = 0.983–0.998, *P* = 0.012) and normal glomeruli (*OR* = 0.978, *95% CI* = 0.964–0.992, *P* = 0.003) were negatively correlated with hyperuricemia. By multivariate logistic regression analysis, only serum creatinine (*OR* = 1.005, *95% CI* = 1.001–1.009, *P* = 0.008) remained to be significantly associated with hyperuricemia (Table 3).

**Table 3:** Logistic regression analysis of factors related to a clinical diagnosis of ANCA-related renal vasculitis patients with hyperuricemia.

Parameter	Univariate analysis		Multivariate analysis	
	OR (95%CI)	*P* value	OR (95%CI)	*P* value
Systolic BP (mmHg)	1.015 (1.003–1.027)	0.015	0.999 (0.979–1.019)	0.902
Diastolic BP (mmHg)	1.037 (1.003–1.072)	0.033	1.035 (0.982–1.091)	0.194
Serum creatinine (µmol/L)	1.004 (1.002–1.006)	0.001	1.005 (1.001–1.009)	0.008
eGFR (ml/min/1.73 m2)	0.977 (0.961–0.994)	0.007	1.017 (0.987–1.047)	0.273
Total cholesterol (mmol/L)	1.457 (1.104–1.923)	0.008	1.354 (0.941–1.935)	0.096
Triglycerides (mmol/L)	1.943 (1.091–3.460)	0.024	1.334 (0.290–2.580)	0.391
CRP (mg/L)	0.990 (0.983–0.998)	0.012	0.994 (0.985–1.003)	0.159
Normal glomeruli (%)	0.978 (0.964–0.992)	0.003	0.975 (0.948–1.002)	0.074
Crescent (%)	1.013 (1.000–1.027)	0.049	0.975 (0.947–1.003)	0.082
Fibrous crescent (%)	1.021 (1.008–1.035)	0.002	1.020 (0.997–1.043)	0.083
Tubular atrophy integral	1.555 (1.003–2.409)	0.048	0.955 (0.500–1.824)	0.888
Interstitial fibrosis integral	1.428 (1.046–1.948)	0.025	0.943 (0.609–1.459)	0.791

### Predictors of patient outcome of ANCA-related renal vasculitis patients with hyperuricemia

**Figure 2:**
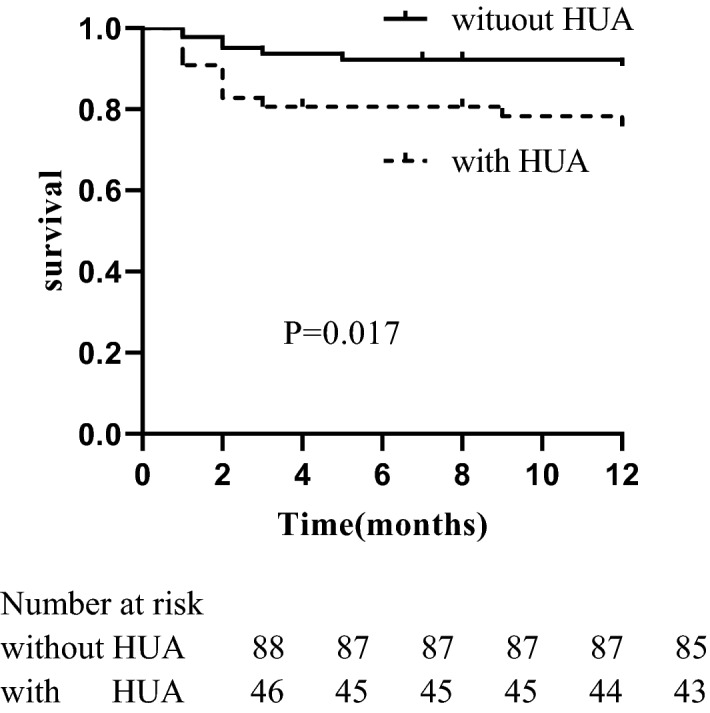
Kaplan–Meier survival curve for ANCA-related renal vasculitis patients.

Over the follow-up 12 months, 26/147 patients (17.69%) were lost to follow-up, and entered the survival analysis as censored dates. 19/142 (12.93%) patients died during the follow-up period, including 7 (36.84%) cases without hyperuricemia and 12 (63.16%) with hyperuricemia. The common causes of death in patients without hyperuricemia were pulmonary infection (3/7, 42.86%), acute cerebral infarction (3/11, 28.57%), alimentary tract hemorrhage (1/7, 14.26%) and lung hemorrhage (1/7, 14.26%), respectively. The common causes of death in patients with hyperuricemia were pulmonary infection (6/12, 50.00%), lung hemorrhage (3/12, 25.00%), acute cerebral infarction (1/12, 0.83%), alimentary tract hemorrhage (1/12, 0.83%) and encephalorrhagia (1/17, 5.88%), respectively. The Kaplan–Meier survival curve in Fig. 2 indicated that hyperuricemia was a predictor of poor prognosis (*P* = 0.017).

Univariate Cox proportional hazard regression model analysis showed that systolic blood pressure (*HR* = 1.017, *95% CI* = 1.002–1.032, *P* = 0.031), diastolic blood pressure (*HR* = 1.043, *95% CI* = 1.002–1.087, *P* = 0.041), serum uric acid (*HR* = 1.008, *95%CI* = 1.004–1.012, *P* < 0.001), the proportion of fibrous crescent (*HR* = 1.016, *95% CI* = 1.000–1.032, *P* = 0.050) and interstitial fibrosis integral (*HR* = 1.535, *95% CI* = 1.025–2.300, *P* = 0.038) were associated with mortality. Multivariate Cox proportional hazard regression model analysis revealed that only serum uric acid (*HR* = 1.007, *95%CI* = 1.002–1.011, *P* = 0.002) was an independent risk factor for patient survival in ANCA-related renal vasculitis patients (Table 4).

**Table 4:** Cox proportional hazard regression model describing long-term risk of death of ANCA-related renal vasculitis patients.

Parameter	Univariate analysis		Multivariate analysis	
	HR (95%CI)	*P* value	HR (95%CI)	*P* value
Systolic BP (mmHg)	1.017 (1.002–1.032)	0.031	1.003 (0.982–1.024)	0.812
Diastolic BP (mmHg)	1.043 (1.002–1.087)	0.041	1.019 (0.967–1.073)	0.478
Serum creatinine (µmol/L)	1.002 (0.999–1.004)	0.138		
eGFR (ml/min/1.73 m^2^)	0.984 (0.961–1.007)	0.179		
Uric acid (µmol/L)	1.008 (1.004–1.012)	< 0.001	1.007 (1.002–1.011)	0.002
Total cholesterol (mmol/L)	1.357 (0.977–1.887)	0.069	1.153 (0.818–1.626)	0.417
Triglycerides (mmol/L)	1.136 (0.600–2.152)	0.695		
CRP (mg/L)	0.995 (0.985–1.005)	0.343		
Normal glomeruli (%)	0.980 (0.959–1.001)	0.062	0.988 (0.961–1.016)	0.411
Crescent (%)	1.008 (0.990–1.026)	0.393		
Fibrous crescent (%)	1.016 (1.000–1.032)	0.050	1.005 (0.987–1.023)	0.596
Tubular atrophy integral	1.205 (0.691–2.101)	0.510		
Interstitial fibrosis integral	1.535 (1.025–2.300)	0.038	1.220 (0.741–2.009)	0.435

### Predictors of renal outcome of ANCA-related renal vasculitis patients with hyperuricemia

**Figure 3:**
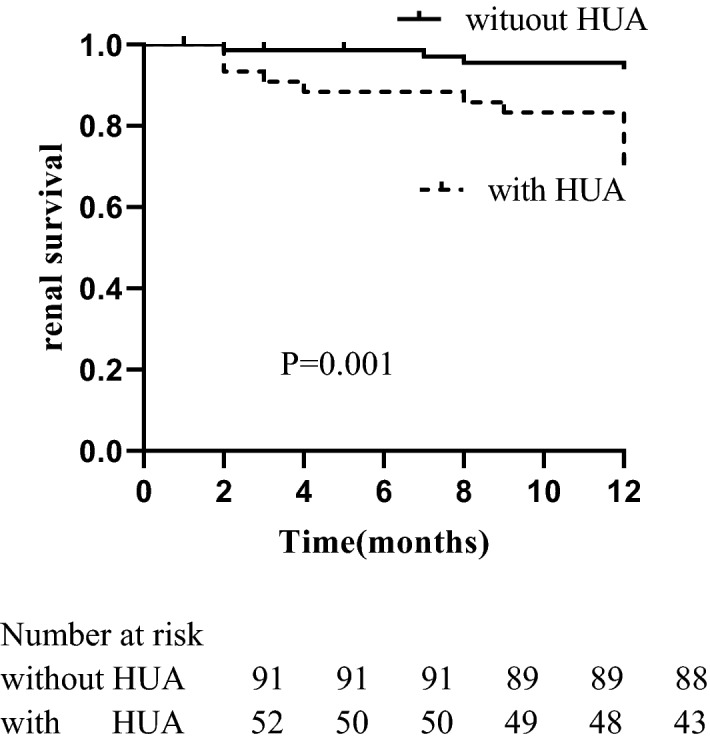
Kaplan–Meier renal survival for ANCA-related renal vasculitis patients.

Among the 147 patients for analysis, 3/92 (3.26%) patients without hyperuricemia and 10/55 (18.18%) with hyperuricemia progressed to ERSD, respectively. Furthermore, 16 patients died before entering ESRD, including 6 cases without hyperuricemia and 10 cases with hyperuricemia. The Kaplan–Meier survival curve in Fig. 3 indicated that the hyperuricemia was a predictor of poor renal survival (*P* = 0.001).

Univariate Cox proportional hazard regression model analysis revealed that serum uric acid (*HR* = 1.009, *95% CI* = 1.004–1.013, *P* = 0.001), serum creatinine (*HR* = 1.004, *95% CI* = 1.002–1.006, *P* < 0.001), eGFR (*HR* = 0.927, *95% CI* = 0.879–0.978, *P* = 0.005), the proportion of normal glomeruli (*HR* = 0.959, *95% CI* = 0.931–0.988, *P* = 0.006), the proportion of fibrous crescent (*HR* = 1.029, *95% CI* = 1.012–1.045, *P* = 0.001) and tubular atrophy integral (*HR* = 2.524, *95% CI* = 1.507–4.228, *P* < 0.001) were associated with ESRD. Multivariate Cox proportional hazard regression model analysis showed that serum uric acid (*HR* = 1.007, *95% CI* = 1.001–1.013, *P* = 0.016) was an independent risk factors for renal survival in ANCA-related renal vasculitis patients. The proportion of normal glomeruli (*HR* = 0.944, *95% CI* = 0.896–0.996, *P* = 0.034) was a protective factor as shown in Table 5.

**Table 5:** Cox proportional hazard regression model describing long-term risk of ESRD of ANCA-related renal vasculitis patients.

Parameter	Univariate analysis		Multivariate analysis	
	HR (95%CI)	*P* value	HR (95%CI)	*P* value
Systolic BP (mmHg)	1.010 (0.994–1.027)	0.214		
Diastolic BP (mmHg)	1.014 (0.967–1.063)	0.563		
Serum creatinine(µmol/L)	1.004 (1.002–1.006)	< 0.001	1.000 (0.995–1.005)	0.987
eGFR (ml/min/1.73 m^2^)	0.927 (0.879–0.978)	0.005	0.951 (0.879–1.029)	0.212
Uric acid (µmol/L)	1.009 (1.004–1.013)	0.001	1.007 (1.001–1.013)	0.016
Total cholesterol (mmol/L)	0.933 (0.624–1.397)	0.737		
Triglycerides (mmol/L)	1.280 (0.648–2.526)	0.477		
CRP (mg/L)	0.992 (0.980–1.004)	0.169		
Normal glomeruli (%)	0.959 (0.931–0.988)	0.006	0.944 (0.896–0.996)	0.034
Crescent (%)	1.021 (1.000–1.042)	0.052	0.977 (0.939–1.016)	0.242
Fibrous crescent (%)	1.029 (1.012–1.045)	0.001	1.015 (0.983–1.048)	0.350
Tubular atrophy integral	2.524 (1.507–4.228)	< 0.001	1.267 (0.595–2.697)	0.540
Interstitial fibrosis integral	1.537 (0.991–2.384)	0.055	1.037 (0.630–1.707)	0.888

## Discussion

This study investigated the clinicopathological characteristics and prognostic factors of 147 AAV patients with renal involvement. Epidemiological studies had shown that the prevalence of hyperuricemia in the general population was 13.3% (19.4% in male and 7.9% in female) in Mainland China^12^. But the prevalence varied greatly according to demographic, socioeconomic, and geographic factors. In our study, the hyperuricemia prevalence in ANCA-associated renal vasculitis was 37.41% (49.09% in male and 50.01% in female). In accordance with previous studies, age was confirmed as a significant risk factor for hyperuricemia, and the prevalence of hyperuricemia increased from the age of 60 years^13,14^. The population susceptible to AAV is elderly patients, so the prevalence of hyperuricemia in ANCA-associated renal vasculitis patients was higher than general population. However, there was no significant difference between genders even if the prevalence of hyperuricemia was lower in male than female. Whether gender affects the incidence of hyperuricemia with ANCA-associated renal vasculitis remains uncertain, and more clinical research is needed.

Renal disease in AAV is common, occurring in over 80% of patients during the disease course and has been identified as a predictor of mortailty of AAV patients^1^. Meanwhile, there is an increasing attention on the relationship between hyperuricemia and kidney diseases^15^. In our study, the level of serum creatinine was much higher and the eGFR was lower in patients with hyperuricemia than those without hyperuricemia, and the prevalence of hyperuricemia rose in accordance with elevation of CKD stage with 51.35% in CKD G4-5. The serum creatinine was an independent risk factor for hyperuricemia. We also found that hyperuricemia was an independent predictor of poor patients and renal survival over a follow-up 12 months, even after adjustment for the covariates including systolic blood pressure, diastolic blood pressure, serum creatinine, eGFR, total cholesterol, triglycerides, CRP, the proportion of normal glomeruli, crescent, fibrous crescent and so on. Higher level of serum uric acid was significantly associated with a higher risk of progressing to ESRD, and was also an independent risk factor for death. It was probably that ANCA-associated renal vasculitis and high level of serum uric acid led to renal function impairment by directly mechanical damage or indirectly inflammation and cell phenotype inversion, resulting in the reduction of uric acid excretion and the formation of a vicious cycle.

As shown in a previous study, hyperuricemia was associated with hypertension, and was also related to the endothelial dysfunction and development of cardiovascular and kidney disease^16^. In our study, the prevalence of hypertension in the 147 AAV patients was 48.98%. It was also observed that both systolic and diastolic blood pressures were significantly higher in ANCA-associated renal vasculitis patients with hyperuricemia. It is likely explained by following mechanisms: hyperuricemia reduced the synthesis and bioavailability of nitric oxide, and induced oxidative stress and inflammation. Oxidative stress contributed to the increase of the inflammatory response in turn, thus resulting in the development of hypertension^17^.

In this study, the level of total cholesterol and triglycerides was also significantly higher in patients with hyperuricemia in comparison to the patients without hyperuricemia. The possible mechanism was that lipid accumulation and lipotoxicity in the kidney lead to lysosomal dysfunction, impaired autophagic flux, inflammation, and oxidative stress^18^. Elevations in lipid level and uric acid level interacted with each other, leading to the impairment of renal structure and function in AAV patients. Consequently, many factors have been involved in the development of hyperuricemia in ANCA-associated renal vasculitis patients, such as hypertension and hyperlipidemia.

C-reactive protein was an acute-phase protein that raised sharply during the inflammatory response, and was a sensitive indicator of AAV activity and infection^19^. Elevated CRP in AAV patients in remission often indicates disease relapse. However, CRP level varies greatly among different patients at the first diagnosis of AAV^20^. C-reactive protein to serum albumin ratio (CAR) may predict all-cause mortality in AAV patients^21^. In this study, both the levels of CRP in two groups were higher than normal level. We separately performed multivariable analysis with CRP, serum creatinine, systolic blood pressure, diastolic blood pressure, total cholesterol and patients with cardiovascular diseases along with other variables with significance in univariable analysis. Only serum creatinine remained to be significantly associated with hyperuricemia. The level of CRP was much lower in patients with hyperuricemia in comparison to the patients without hyperuricemia. The reason was probably that serum uric acid played an antioxidant and anti-inflammatory role as a powerful oxygen radical scavenger^22,23^. Therefore, high serum uric acid levels led to attenuated inflammatory response, the decreased CRP production and the accelerated plasma clearance in ANCA-associated renal vasculitis patients with hyperuricemia. Due to the interaction between inflammatory response and complement system^19^, raised CRP levels might inhibit alternative complement pathway activation, which played a complex role in the progression of ANCA-associated renal vasculitis with hyperuricemia.

Our study showed that there was no significant differences in age, gender, patients with hypertension at diagnosis, patients with diabetes mellitus at diagnosis, patients with cardiovascular diseases at diagnosis, the level of ESR, hemoglobin, serum albumin, 24 h-proteinuria, urine RBC, complement C3, complement C4, HDL-C, LDL-C, anti-MPO, anti-PR3 and BVAS between patients with and without hyperuricemia. It was likely because patients with different demographic, socioeconomic, and geographic factors were included in this study and the sample size was small.

In clinical work, some patients are unable to undergo kidney biopsy, while biopsy is important for diagnosis and prognosis in AAV. Histologically, pauci-immune necrotizing crescentic glomerulonephritis is the most typical pathological characteristics in ANCA-associated renal vasculitis. Previous studies indicated that severe damage and rupture of the glomerular capillary wall often led to the crescent formation, which was associated with higher risk of renal function and renal progression^24^. Recent study demonstrated that patients developed ESRD directly when the percentage of fibrous crescent + global glomerulosclerosis exceeded 32.6%^25^. Our results showed that the proportion of crescents, fibrous crescents, tubular atrophy integral and interstitial fibrosis integral was higher in ANCA-associated renal vasculitis patients with hyperuricemia than patients without hyperuricemia, and all of them were associated with hyperuricemia occurrence in ANCA-associated renal vasculitis patients. Besides, the proportion of fibrous crescents was associated with patient and renal survival in this study. Tubular atrophy was positively correlated with poor renal outcome, and interstitial fibrosis was associated with poor patient survival. They were not independent predictors. Therefore, they could be used as monitoring indexes for identifying patients at increased risk of developing hyperuricemia, progressing to ESRD and death. We also found that the proportion of normal glomeruli was higher in ANCA-associated renal vasculitis patients without hyperuricemia than patients with hyperuricemia. The proportion of normal glomeruli was a protective factor for hyperuricemia in ANCA-associated renal vasculitis patients, as well as an independent protective factor for renal survival. We concluded that early detection and intervention of hyperuricemia in ANCA-associated renal vasculitis patients may preserve more normal glomeruli and residual renal function. There were no differences in cellular crescent, fibrocellular crescent, fibrinoid necrosis, global glomerulosclerosis, destruction of the basement membrane of Bowman’s capsule, interstitial inflammatory cell infiltration integral and microangiopathic lesions between two groups, which may be due to the small size of the above pathological types in this study.

The limitations of this study were summarized. Firstly, the follow-up period was short and the sample size was also limited. The patients enrolled in this study may not be representative of all ANCA-associated renal vasculitis patients. Secondly, the patients taking antigout drugs within 3 months before admission were excluded, and the effect of therapy on hyperuricemia could not be analyzed.

## Conclusions

In conclusion, ANCA-associated renal vasculitis patients with hyperuricemia presented more severe clinical characteristics and renal pathological features. The level of serum creatinine was an independent risk factor for hyperuricemia. The level of serum uric acid was an independent risk factors for patients and renal survival in ANCA-associated renal vasculitis patients. The proportion of normal glomeruli was an independent protective factor for renal survival. The crescents formation, the proportion of fibrous crescent, tubular atrophy and interstitial fibrosis were likely contributed to severe clinical characteristics and renal pathological changes in ANCA-associated renal vasculitis patients with hyperuricemia. Hyperuricemia has an important influence on the progression of ANCA-associated renal vasculitis. A good control of serum uric acid may improve the prognosis. Randomized controlled studies are needed for this purpose.

## Methods

### Study participants and data collection

**Figure 4:**
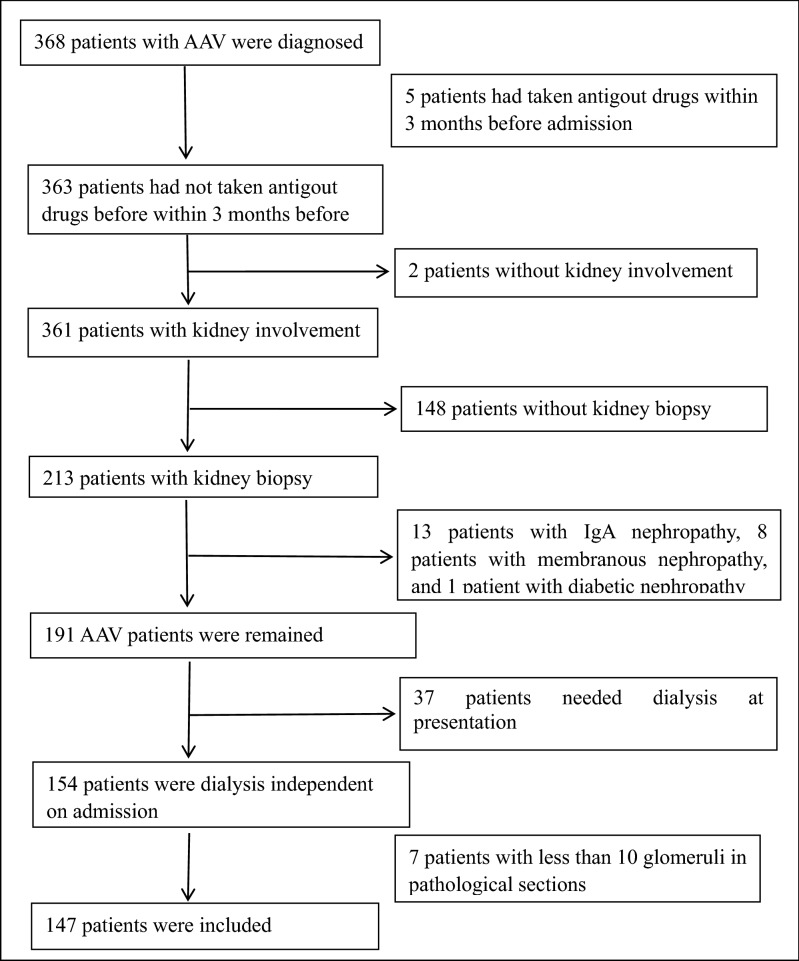
Patients selection flowchart. A total of 368 patients with AAV were diagnosed. 5 patients had taken antigout drugs within 3 months before admission and 2 patients without kidney involvement were excluded. Among remaining 361 patients, 148 patients did not receive kidney biopsy. 13 patients with IgA nephropathy, 8 patients with membranous nephropathy, and 1 patient with diabetic nephropathy were excluded, too. 7 patients whose glomeruli less than 10 glomeruli in pathological sections and 37 patients needed dialysis at presentation were also excluded. At last, 147 patients were included in this study.

Participants of ANCA-associated renal vasculitis receiving renal biopsy were screened in the First Affiliated Hospital of Zhengzhou University from January 2014 to April 2019. The inclusion criteria were (1) renal impairment with proteinuria (> 300 mg/day) and/or haematuria (> 10/mm^3^); (2) a positive ANCA assay (indirect immunofluorescence and/or an antigen-specific immunoassay); (3) a renal biopsy confirming the presence of pauci-immune glomerulonephritis, and ≥ 10 glomeruli were observed in pathological sections; (4) patients did not take antigout drugs within 3 months before admission. The time of diagnosis was defined as the date of the first ANCA-positive assay. The exclusion criteria included systemic diseases involving the kidneys, such as systemic lupus erythematosus, Sjogren's syndrome, hepatitis B virus associated glomerulonephritis, Henoch-Schonlein purpura nephritis and diabetic nephropathy, other primary glomerular diseases, such as IgA nephropathy, membranous nephropathy, etc. Patients needed dialysis on admission were also excluded. 147 patients with ANCA-associated renal vasculitis were enrolled in this study, including 76 males and 71 females. The average age was 59.50 ± 11.66 years old. Participants were followed-up for 12 months, and progressing to the ESRD and death was treated as the endpoint event. This study was approved by the Ethics Committee of the First Affiliated Hospital of Zhengzhou University (Henan, China, No.2019-KY-015) (Fig. 4).

#### Statement

All methods were performed in accordance with the relevant guidelines and regulations.

### Clinical and laboratory findings

Clinical data included the following: gender, age, hypertension, diabetes mellitus, cardiovascular diseases, serum creatinine, serum uric acid, estimated glomerular filtration rate (eGFR), systolic blood pressure, diastolic blood pressure, hemoglobin, serum albumin, 24 h-proteinuria, urine RBC counts, CRP, ESR, complement C3, complement C4, total cholesterol, triglycerides, high-density lipoprotein cholesterol (HDL-C), low-density lipoprotein cholesterol (LDL-C), myeloperoxidase antibody (anti-MPO), protease 3 antibody(anti-PR3), Birmingham Vasculitis Activity Score (BVAS). Blood samples were taken for laboratory test. eGFR was calculated with the Chronic Kidney Disease Epidemiology Collaboration (CKD-EPI) equation^26^. The diagnosis criteria for hyperuricemia are defined as serum uric acid levels, male > 420 μmol/L (7 mg/dL), female > 357 μmol/L (6 mg/dL)^27^. Referring to the 2009 U.S. KDIGO guidelines, CKD is clinically divided into 5 stages according to eGFR level^28^. Hypertension referred to a blood pressure ≥ 140/90 mmHg. The blood pressure measurements were repeated twice in a patient in a sitting position and in the patient’s right arm^29^. Diabetes mellitus was defined by the American Diabetes Association (ADA) "Standards of Medical Care in Diabetes-2019". Participants were identified as having diabetes mellitus if they had FPG ≥ 126 mg/dL (7.0 mmol/L) or 2-h PG ≥ 200 mg/dL (11.1 mmol/L) during a 75-g oral glucose tolerance test (OGTT) or HbA1c ≥ 6.5% (48 mmol/mol) or a random plasma glucose ≥ 200 mg/dL (11.1 mmol/L) in a patient with classic symptoms of hyperglycemia or hyperglycemic crisis^30^. Cardiovascular diseases are mostly caused by atherosclerosis, and the most common are coronary artery disease and hypertensive heart disease. Coronary artery disease refers to typical angina symptoms, ischemic ST-T changes in electrocardiograms, or a history of myocardial infarction. Asymptomatic patients are identified as having coronary artery disease if they have ischemic ST-T changes in electrocardiograms or are positive in exercise tests^31^. The diagnostic criteria for high heart disease are defined as left ventricular high voltage or ST-T changes in electrocardiogram or left ventricular hypertrophy in echocardiogram^32^.

### Histological examination of renal biopsy specimens

Renal specimens were obtained by percutaneous needle biopsy and were routinely examined by light microscopy, immunofluorescence and electron microscopy. The presence of crescent, cellular crescent, fibrocellular crescent, fibrous crescent, fibrinoid necrosis, global glomerulosclerosis and destruction the basement membrane of Bowman's capsule of each glomerulus was recorded and calculated as the percentage of the total number of glomeruli. The semi-quantitative scoring system was used to analyze tubulointerstitial lesions. The semi-quantitative criteria for interstitial inflammation is evaluated according to the extent of inflammatory cells in the cortex: 0 = no or trivial interstitial inflammation (< 10% of unscarred parenchyma), 1 = 10–25% of parenchyma inflamed, 2 = 26–50% of parenchyma inflamed, 3 = more than 50% of parenchyma inflamed. Interstitial fibrosis and tubular atrophy are evaluated according to the extent of interstitial fibrosis with tubular atrophy in the cortex: 0 = no or trivial interstitial fibrosis (< 5% of unscarred parenchyma), 1 = 6–25% of interstitial fibrosis, 2 = 26–50% of interstitial fibrosis, 3 = more than 50% of interstitial fibrosis. The presence of microangiopathic lesions was calculated as the percentage of the number of renal specimens in two groups, respectively^33^. (1) Normal glomeruli are defined as the presence of mild changes due to ischemia or a small amount of inflammatory cell infiltration (less than four neutrophils, lymphocytes or monocytes) while the absence of vasculitis lesions and bulbar sclerosis. (2) Global glomerulosclerosis is defined as a sclerotic area with scarring involving more than 50% glomerular tuft. Cellular crescent is defined when more than 50% crescent is occupied by the cells. (3) Fibrous crescent is defined when more than 90% crescent is occupied by the extracellular matrix. (4) The fibrocellular crescent is defined when less than 50% crescent is occupied by cells and less than 90% of the crescent is occupied by the extracellular matrix. (5) Tubular atrophy is defined by a thick irregular TBM with decreased diameter of the tubules. (6) Interstitial inflammatory cell infiltration is defined as an excess of inflammatory cells within the cortical interstitium, excluding the subcapsular area and the area of surrounding global glomerulosclerosis. (7) Interstitial fibrosis is defined as increased extracellular matrix separating tubules in the cortical area, excluding the subcapsular area. (8) Microangiopathic lesions including endothelial cell swelling and subintimal edema and arteriolar thrombosis and/or fibrinoid necrosis were considered acute. The arterial “onion-skin” lesions (fibrous intimal thickening with concentric lamination) were considered chronic^33^.

### Statistical analyses

Consecutive variables were expressed as the mean ± standard deviation or median (interquartile range [IQR]). Categorical variables were presented as frequency and percentages and tested with Fisher and χ^2^ test. The logistic regression model was used for analyzing the influencing factors of hyperuricemia in AAV with renal injury patients. Kaplan–Meier analysis was used to analyze patient and renal survival. Univariate and multivariate cox regression models were used for identifying the predictors of patient and renal outcomes. The results were expressed as hazard ratios (HRs) (with 95% confidence interval [CI]). *P* < 0.05 was considered as statistical significance. SPSS 23.0 (IBM Corp., Armonk, NY, USA) was used for all the statistical analyses. GraphPad Prism 8.0.2 (GraphPad Software, Inc., San Diego, CA, USA) was used to plot survival curves.

### Informed consent

The informed consent was obtained from all subjects (above 18 years old) and the informed consent was also obtained from parent and/or legal guardian of participants less than 18 years old.
